# Liquid phase exfoliation of talc: effect of the medium on flake size and shape

**DOI:** 10.3762/bjnano.14.8

**Published:** 2023-01-09

**Authors:** Samuel M Sousa, Helane L O Morais, Joyce C C Santos, Ana Paula M Barboza, Bernardo R A Neves, Elisângela S Pinto, Mariana C Prado

**Affiliations:** 1 Campus Santa Luzia, Instituto Federal de Minas Gerais, R. Érico Veríssimo, 317, Santa Luzia – MG, 33115-390, Brazil; 2 Centro de Desenvolvimento da Tecnologia Nuclear CDTN/CNEN, Avenida Antônio Carlos, 6627, Belo Horizonte, Minas Gerais 31270-901, Brazil; 3 Physics Department, Universidade Federal de Ouro Preto, R. Diogo de Vasconcelos, 122, Ouro Preto – MG, 35400-000, Brazil; 4 Physics Department, Universidade Federal de Minas Gerais, Avenida Antônio Carlos, 6627, Belo Horizonte – MG, 31270-901, Brazil; 5 Campus Ouro Preto, Instituto Federal de Minas Gerais, R. Pandiá Calógeras, 898, Ouro Preto – MG, 35400-000, Brazil

**Keywords:** 2D materials, atomic force microscopy, liquid phase exfoliation, nanomaterials, talc

## Abstract

Industrial applications of nanomaterials require large-scale production methods, such as liquid phase exfoliation (LPE). Regarding this, it is imperative to characterize the obtained materials to tailor parameters such as exfoliation medium, duration, and mechanical energy source to the desired applications. This work presents results of statistical analyses of talc flakes obtained by LPE in four different media. Talc is a phyllosilicate that can be exfoliated into nanoflakes with great mechanical properties. Sodium cholate at two different concentrations (below and at the critical micelar concentration), butanone, and Triton-X100 were employed as exfoliation medium for talc. Using recent published statistical analysis methods based on atomic force microscopy images of thousands of flakes, the shape and size distribution of nanotalc obtained using the four different media are compared. This comparison highlights the strengths and weaknesses of the media tested and hopefully will facilitate the choice of the medium for applications that have specific requirements.

## Introduction

Two-dimensional (2D) materials have attracted a lot of interest due to their outstanding properties [1]. However, large-scale production is still a challenge that needs to be addressed to integrate 2D materials into industrial applications. One approach to producing large quantities of few-layer flakes of a broad range of exfoliatable materials is liquid-phase exfoliation (LPE) [2–5]. This method relies on mechanical energy to exfoliate materials in an appropriate liquid medium. To exfoliate a material of interest, it must be reduced to a fine powder and mixed with a liquid that serves as an exfoliation medium. The solution is exposed to a mechanical energy source that leads to the delamination of the material, resulting in a suspension of nanosheets [6]. The energy may be provided by an ultrasonic bath, a shear force mixer, or a tip sonicator. The solution serves three purposes: it provides a medium to propagate the mechanical energy, suspends the exfoliated nanosheets, and prevents them from agglomerating again. The versatility of the method allows it to be employed to obtain nanoflakes of a collection of materials such as graphene [3,7], hexagonal boron nitride [8], transition metal dichalcogenides [9], and others [10–11].

Although the experimental setup is generally designed as described before [6], numerous parameters must be adjusted to optimize the exfoliation for a given material and the available experimental setup. The exfoliation medium must be chosen correctly to guarantee the optimum result. And one must keep in mind that completely separating the nanoflakes from the solution might not be an easy task, if possible at all. So, if the flakes can be obtained in an application-friendly medium, it will greatly facilitate the process.

In this study, we addressed the implications of the choice of medium for shape and size of talc nanoflakes obtained by LPE. This material is a hydrated magnesium silicate belonging to the phyllosilicate group [12]. Phyllosilicates are crystalline minerals with a basic Si_2_O_5_ composition that exhibit a layer structure, making them ideal candidates for mechanical exfoliation. Talc already has several industrial applications [12], ranging from polymer and ceramics fillers [13–16] to pharmaceutical and cosmetics uses [17]. It was shown that monolayer talc has outstanding mechanical properties of the same order of magnitude as graphene [12]. The breaking strength for uniaxial deformations ranges from 29 to 33 N·m^−1^, and the two-dimensional elasticity modulus is *E* = 181 N·m^−1^. Also, talc’s flexural rigidity is about three times that of graphene but it can be bent to small curvatures without fracturing. These properties make nanoscale talc a promising candidate for the application [14–15,18] as reinforcement for polymers and other composites, including biocompatible materials, and van der Waals heterostructures. Being able to scale the production is a crucial step to realizing applications at an industrial level. We present the results of liquid-phase exfoliation of talc using different liquid media, namely sodium cholate aqueous solution (6 mg/mL and 1 mg/mL), Triton X-100 aqueous solution, and butanone. The mechanical energy necessary to delaminate the mineral was provided by an ultrasonic bath. We report a statistical analysis of the dimensions (measured by atomic force microscopy) of the nanoflakes obtained employing the four routes, evidencing that the exfoliation medium has an important influence on flake size and shape and should be accounted for when designing a production route with the desired application in mind.

## Results and Discussion

### Choice of exfoliation medium

Four exfoliation media were employed in this work, as summarized in Table 1. The first one was an aqueous solution of sodium cholate (SC) at 6 mg/mL, previously employed in the literature [11]. SC is a bile salt ionic surfactant widely employed in LPE [6,19–21]. While it is less toxic than other organic compounds usually employed for the same purpose, such as *N*-methyl-pyrrolidone (NMP), it is expensive and can leave residues on exfoliated flakes. Although fundamental to LPE, the role of the concentration and chemical composition of the exfoliation medium is still not fully understood [6]. Bearing that in mind, we also tested SC at a much lower concentration of 1 mg/mL. The critical micelle concentration (CMC) of SC at room temperature ranges from 5.2 to 6.5 mg/mL [22]. The dilute solution is guaranteed to be well below the CMC, which is regarded as preferable [6]. To guarantee that the effects seen in flake size after exfoliation were not due to a change in the relative concentration of SC to talc, we also added less talc powder to the solution, to keep the ratio constant. We also tested the nonionic surfactant Triton-X100. Besides the absence of charged groups, compared to SC, Triton-X100 is also less expensive, although not environmentally friendly either. Finally, we tested an organic solvent, namely butanone. Butanone is volatile and has a boiling point of approximately 80 °C, making it the easiest medium to remove after exfoliation of the four employed here. Also, unlike other organic solvents commonly used in LPE, for example, NMP or dimethylformamide (DMF), that have a higher boiling point, butanone leaves less residues when exfoliated flakes are deposited onto substrates for atomic force microscopy (AFM) measurements. Table 1 summarizes the solutions tested here, and details of the sample preparation can be found in the Experimental section.

**Table 1:** Initial concentration of talc and surfactants/organic solvents for exfoliation.

Sample	Exfoliation medium	Talc concentration

SC6	sodium cholate/DI water 6 mg/mL	6 mg/mL
SC1	sodium cholate/DI water 1mg/mL	1 mg/mL
Triton	Triton-X100/DI water 1 mg/mL	6 mg/mL
butanone	butanone (pure)	6 mg/mL

### Liquid exfoliation of talc

**Figure 1:**
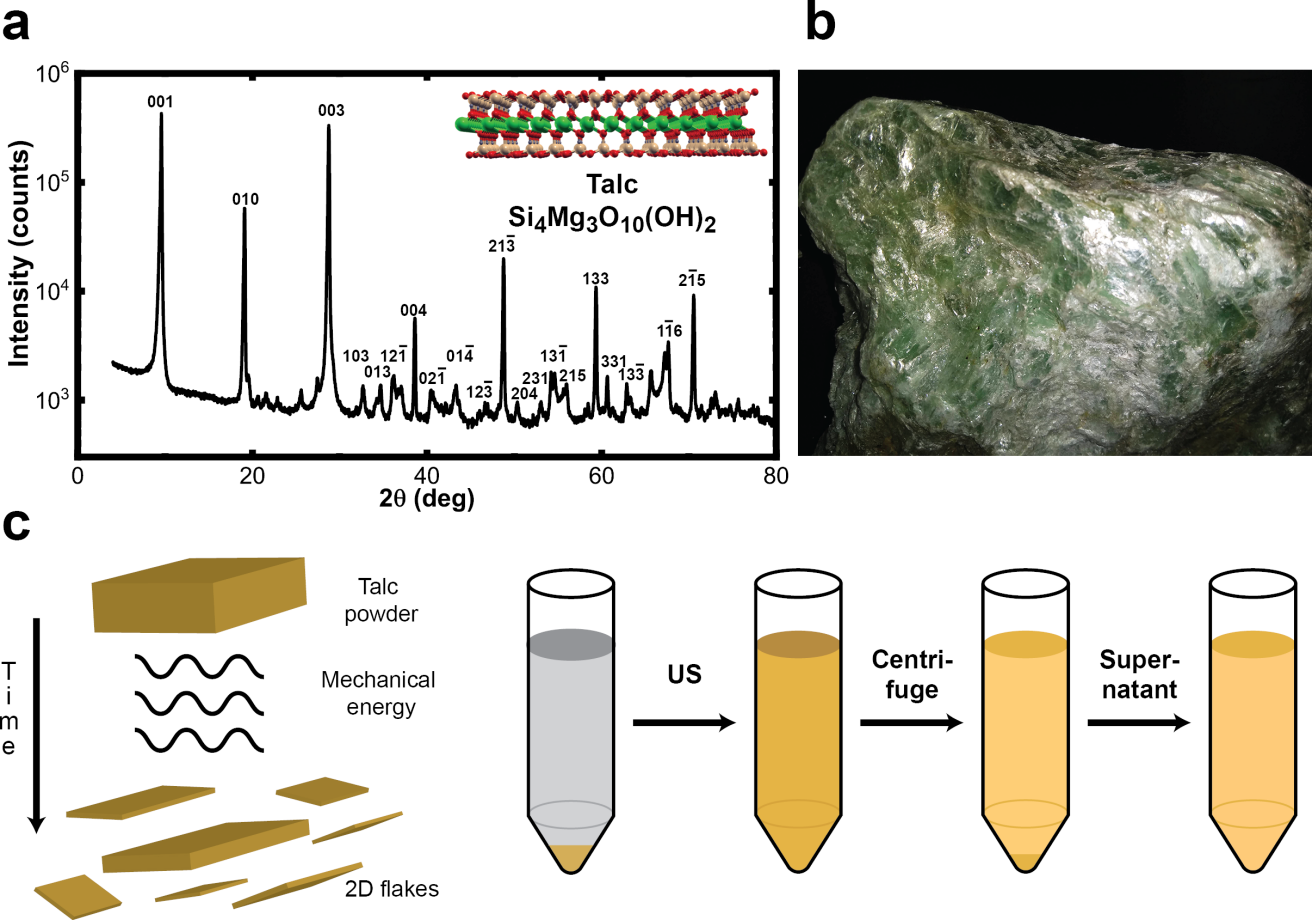
Talc characterization and exfoliation procedure. (a) XRD data for the sample employed here. The insert shows formula and structure of talc [12]. Pink, red, green, and gray circles represent Si, O, Mg, and H atoms, respectively. (b) Picture of the source mineral before being manually milled. It weighed approximately 1.3 kg. (c) Schematic representation of LPE. Micrometer-sized talc powder is exposed to mechanical energy, which leads to delamination of its layers. After exfoliation, the sample is centrifuged to separate non-exfoliated flakes from nanometer-sized flakes, and the supernatant is collected for further analysis.

Talc powder was exfoliated in each liquid medium by exposure to mechanical energy provided by an ultrasonic bath (full details in the Experimental section). Talc was manually milled down to a fine powder and characterized by X-ray diffraction (XRD). Figure 1a displays the results. All peaks are assigned to talc, when compared with the crystallographic database, and many are labeled. The insert shows the structure and chemical formula of talc [23]. Figure 1b shows the mineral that was milled to a fine powder. The powder was mixed with the exfoliation medium and subjected to mechanical energy provided by an ultrasonic bath (Figure 1c).

Centrifugation was performed to separate non-exfoliated material from nanometer-sized flakes. To analyze the influence of the exfoliation medium on shape and size of the nanoflakes obtained by LPE, we carefully chose acceleration and duration of the centrifugation. Thick flakes (>100 nm) must be removed to implement a semi-automated analysis of thousands of flakes based on AFM images that provide a robust statistical representation of the sample [24–25]. At the same time, the removal of flakes that are few to tens of nanometers thick would make the effect of exfoliation medium on size and shape less evident. Therefore, a single centrifugation step of one hour only at 1000*g* was employed. Such low acceleration will not produce a monolayer-rich solution [26], which is crucial for the analysis we aim to perform.

### Atomic force microscopy characterization of flake size

**Figure 2:**
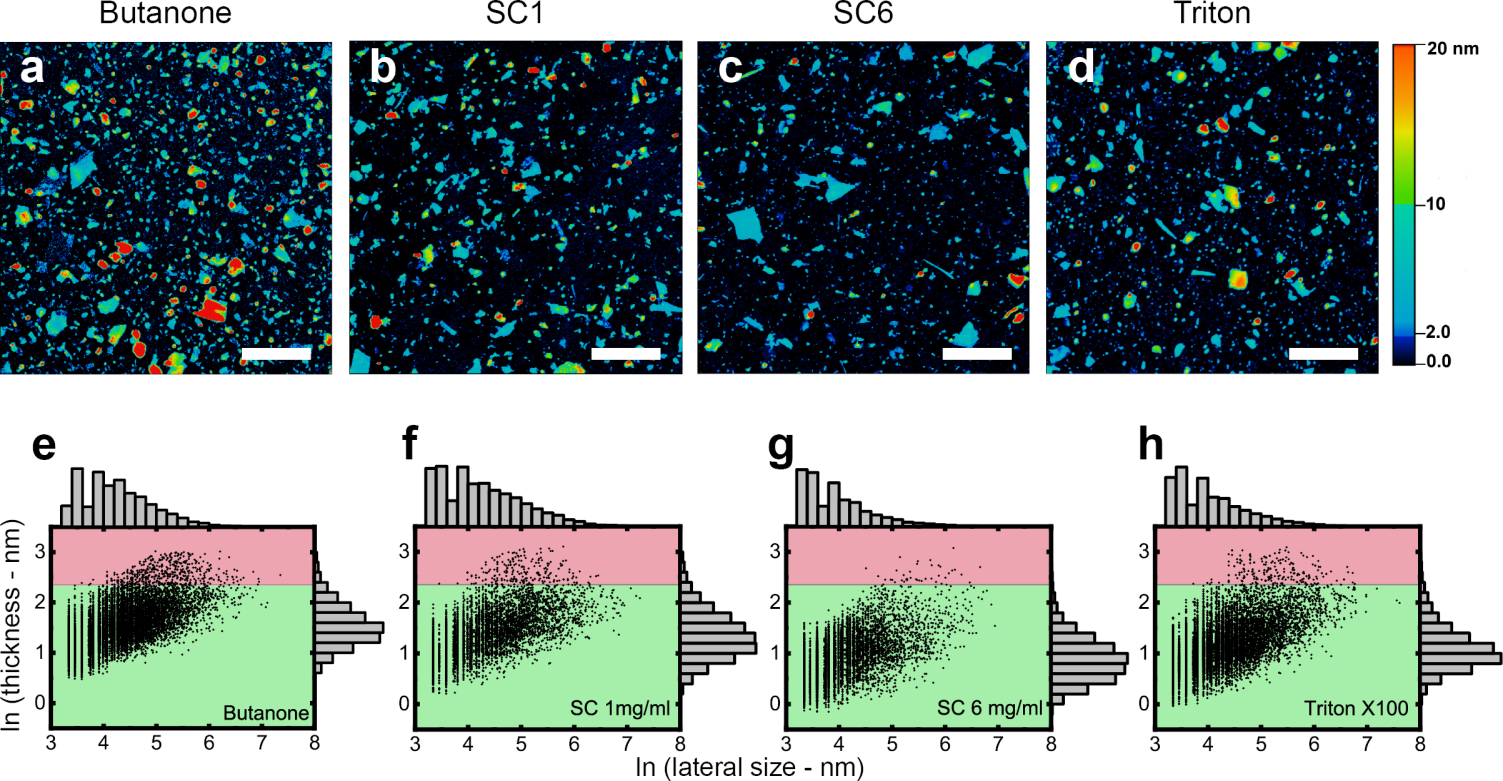
Atomic force microscopy images of the samples produced employing the four different media under investigation. (a–d) AFM images of samples expoliated with butanone, sodium cholate at 1 mg/mL, sodium cholate at 6 mg/mL, and triton-X100, respectively. The scale bars are 1 μm. (e–h) Statistics from the four different samples in the same order as the AFM images. The top histograms indicate the lateral size distribution, whereas the side histograms show the thickness distribution. The green region marks flakes that are less than 10.5 nm thick while pink represents flakes thicker than 10.5 nm.

Figure 2 shows the results obtained for each exfoliation medium. Figure 2a–d shows AFM topographical images of samples exfoliated in butanone, SC1, SC6, and Triton-X100, respectively. A single vertical scale was chosen to facilitate the visualization of flakes of different thicknesses in all samples. The substrate appears in black to dark blue. Following previous works, we consider flakes with ten or less layers as “few-layer” [25]. Since talc has a layer thickness of approximately 1 nm [12], we did not convert the height to the number of layers as it is a direct conversion. Few-layer flakes appear in light blue. Flakes that are thicker than 10 nm and thinner than 20 nm appear in green, yellow, and orange shades. Red represents everything of 20 nm thickness or thicker.

It is easy to see that all four exfoliation media produced samples mainly consisting of few-layer flakes (thinner than 10.5 nm, accounting for a thicker first layer and/or exfoliation medium residue [6,24]). At the same time, even without an in-depth analysis, it is clear that the medium has a very important influence on the flake size distribution. The sample exfoliated in sodium cholate at 6 mg/mL has visually fewer flakes in the pink region of the distribution graphics (Figure 2e–h). Butanone seems to have yielded a less dispersed distribution of flakes, although with higher thicknesses than other media.

Table 2 provides statistical parameters for the four samples. To characterize the lateral size of the flakes, the so-called Feret diameter was employed (the maximum Feret diameter of a flake, *F*, is the largest distance between two parallel tangential lines in any in-plane direction of a flake) [27]. It would be simple to conclude that sodium cholate at 6 mg/mL produces the sample with the smallest mean flake thickness (*h*) and lateral size. If one desires a sample aimed at an application where flake thickness is critical and monolayers and bilayers are preferable, at a first glance, this would be the SC6 sample. However, much more information can be obtained using the procedures proposed by the authors of [24].

**Table 2:** Comparison of four different talc LPE samples. The total number of flakes analyzed, mean height (*h*) and its standard deviation (σ*_h_*), mean lateral size (Feret diameter, *F*), and its standard deviation (σ*_F_*), are shown.

Medium	No.	<*h*> (nm)	σ*_h_* (nm)	<*F*> (nm)	σ*_F_* (nm)

But.	11458	5.2	2.5	90	84
SC1	6286	4.5	2.6	100	106
SC6	8405	2.7	1.5	60	71
Triton	16494	3.4	2.0	77	84

As discussed by Fernandes and co-workers [24], simply looking at mean flake thickness and standard deviation of a sample does not account well for the volume (or mass) of few-layer flakes versus bulk flakes (thicker than 10.5 nm for talc, which represents 10 or more layers). We calculated the mass ratio of bulk (*M*) and few-layer (*m*) flakes. This ratio is defined as follows:


[1] $$\frac{M}{m}=\frac{p_{\text{n}}}{p_{\text{v}}}\frac{\left(1-p_{\text{v}}\right)}{\left(1-p_{\text{n}}\right)},$$

where *p*_n_ is the probability of a randomly picked flake being a few-layer flake and *p*_v_ is the volume fraction of few-layer flakes. See Supporting Information of [24] for details on the calculation.

The sample with the smallest *M*/*m* ratio is SC1 (5.6), followed by butanone (11.6) and Triton-X100 (16.6). Surprisingly, the sample with the highest ratio is SC6 (40.5). This can be understood in light of the meaning of the mass ratio. For every few-layer flake in the SC6 sample, the bulk flakes will correspond to a mass of ca. 41 few-layer flakes. Since the few-layer flakes are very small in this sample (thickness and Feret diameter), a bulk flake weights the same as many small flakes. This has serious implications for applications that demand few-layer flakes.

Centrifugation at higher accelerations can remove bulk flakes changing the parameters obtained here. An interesting hypothesis discussed in recent works [25,28] is that centrifugation might also lead to the loss of the smallest flakes along with the large ones due to drag effects. Flakes of the SC6 sample would be very susceptible to this effect, and a single centrifugation step at high acceleration should be avoided.

### Topological vector analysis

**Figure 3:**
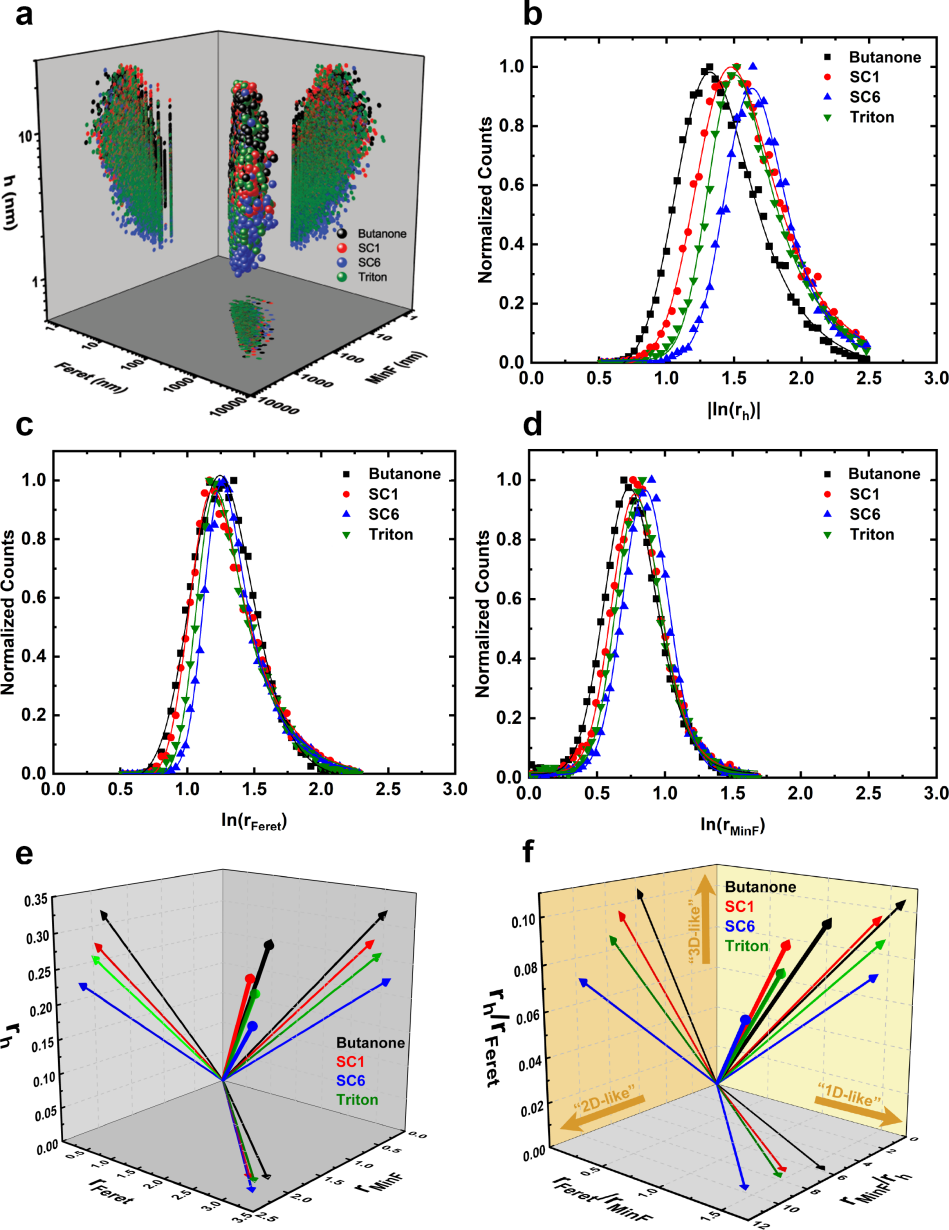
Characteristic lengths, normalized probability distributions, and topological vectors of the dimensionless aspect ratios *r_h_*, *r*_Feret_, and *r*_MinF_ for talc flakes obtained via LPE in four different media. (a) 3D plot of average height and maximum and minimum Feret diameters for all flakes of the four samples. (b–d) Normalized distributions of ln(*r_h_*), ln(*r*_Feret_) and ln(*r*_MinF_), respectively, for each sample (black squares: butanone; red circles: sodium cholate at 1 mg/mL; blue upward triangles: sodium cholate at 6 mg/mL; green downward triangles: Triton-X100). For *r_h_*, the absolute value of the logarithm was plotted to avoid negative values and facilitate comparison with the other parameters. (e) Three-dimensional topological vector representation of *r_h_*, *r*_Feret_, and *r*_MinF_ for all samples. (f) Topological vectors of three pairwise ratio combinations of *r_h_*, *r*_Feret_, and *r*_MinF_.

To further investigate the differences between samples exfoliated in different media, we use now the methodology proposed in [25]. Figure 3a shows a 3D graphic representation of all the flakes in the four samples (several thousand flakes were analyzed for each case). We characterize size and shape of each flake considering its average thickness (*h*), maximum Feret diameter (Feret), and minimum Feret diameter (MinF, the smallest distance between two tangential parallel lines in any in-plane direction of a flake [27]). Recapping the discussion made by the authors who also use some of the methodology proposed by Chacham and colleagues [28], using AFM data we calculate three dimensionless aspect ratios:


[2] $$r_{\text{h}}=\frac{h}{\sqrt[{3}]{V}},\;r_{\text{Feret}}=\frac{\text{Feret}}{\sqrt[{3}]{V}},\;r_{\text{MinF}}=\frac{\text{MinFeret}}{\sqrt[{3}]{V}}.$$

Next, we plotted the probability distributions of the logarithm of these aspect ratios, ln(*r_h_*), ln(*r*_Feret_), and ln(*r*_MinF_). Figure 3b–d shows the distribution histograms of these values for all four different exfoliation media under investigation here. The distributions are skewed and best described by the exponentially modified Gaussian (EMG) distribution [25]. The probability density function of the EMG distribution is given by:


[3]

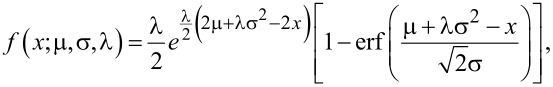



where μ and σ are the mean and the variance of the Gaussian distribution and λ is the exponential decay rate; erf(*x*) is the error function. The applicability of this distribution and further details are discussed in a previous work [25].

As can be seen in Figure 3b–d, an EMG function fitted the data very well with *R*^2^ > 0.99. The thickness parameter distribution is the widest one for all samples while the minimum lateral diameter (MinFeret) one is the narrowest and most symmetrical of all three parameters. This was observed and discussed before for talc and graphene samples [25].

Using the most probable value (mode) as the representative value for each of the three dimensionless aspect ratios, we constructed the topological vector representation of each sample (Figure 3e). This representation is very useful since it immediately brings out the differences between each sample. The projections of the vector in the planes readily offer a comparison of the characteristics of the sample. The samples differ more in thickness-related parameters (thus, the number of layers) than in the lateral size-related parameters.

To further compare shape-related features of the samples, we plotted topological vectors of pairwise ratios among these components (Figure 3f) [26]. The arrows in Figure 3f emphasize the meaning of the ratios. The *r*_h_/*r*_Feret_ axis correlates with the voluminosity of a flake, that is, a greater the value indicates a more three-dimensional shape of the sample. A greater value of the *r*_Feret_/*r*_MinF_ axis indicates a more ribbon-shaped flake, that is, a more one-dimensional shape. Finally, greater values of the *r*_MinF_/*r*_h_ axis indicate a more plate-shaped flake, that is, a more two-dimensional shape.

The four media investigated here clearly produce samples with different shape characteristics. SC6 flakes are the most two-dimensional, while those exfoliated with butanone are the least two-dimensional, having a more 3D shape than all other samples. It is interesting to note that the SC1 and Triton-X100 samples are very alike. The dilution of sodium cholate has an important influence on the shape of the exfoliated flakes. Since the talc concentration was also diminished to keep the mass ratio between surfactant and talc constant, the effect must be due to the surfactant arrangement (i.e., the presence or absence of molecular aggregates) and a higher relative amount of dispersion medium (the water-to-talc ratio is larger in the SC1 sample).

As stressed by Santos and colleagues [25], shape and size are different things. The previous analysis of bulk versus few-layer flakes is very important to complement the topological vectors just discussed because having a 2D shape does not mean that the flake has few layers. A bulk flake that has a thickness much smaller than both lateral parameters is 2D-shaped but behaves like the bulk material and not like mono-layer or few-layer flakes.

Adding to the discussion, let us consider the asymmetry of the distribution curve for each sample. A Gaussian distribution is symmetric while the EMG is not; it is possible to characterize this asymmetry by calculating two shape parameters, *k* (asymmetry) and τ (trimness), both functions of σ and λ:


[4] $$k={\left(\sigma \lambda \right)}^{-1}$$

[5] $$\tau =\frac{\sigma }{\lambda }$$

**Figure 4:**
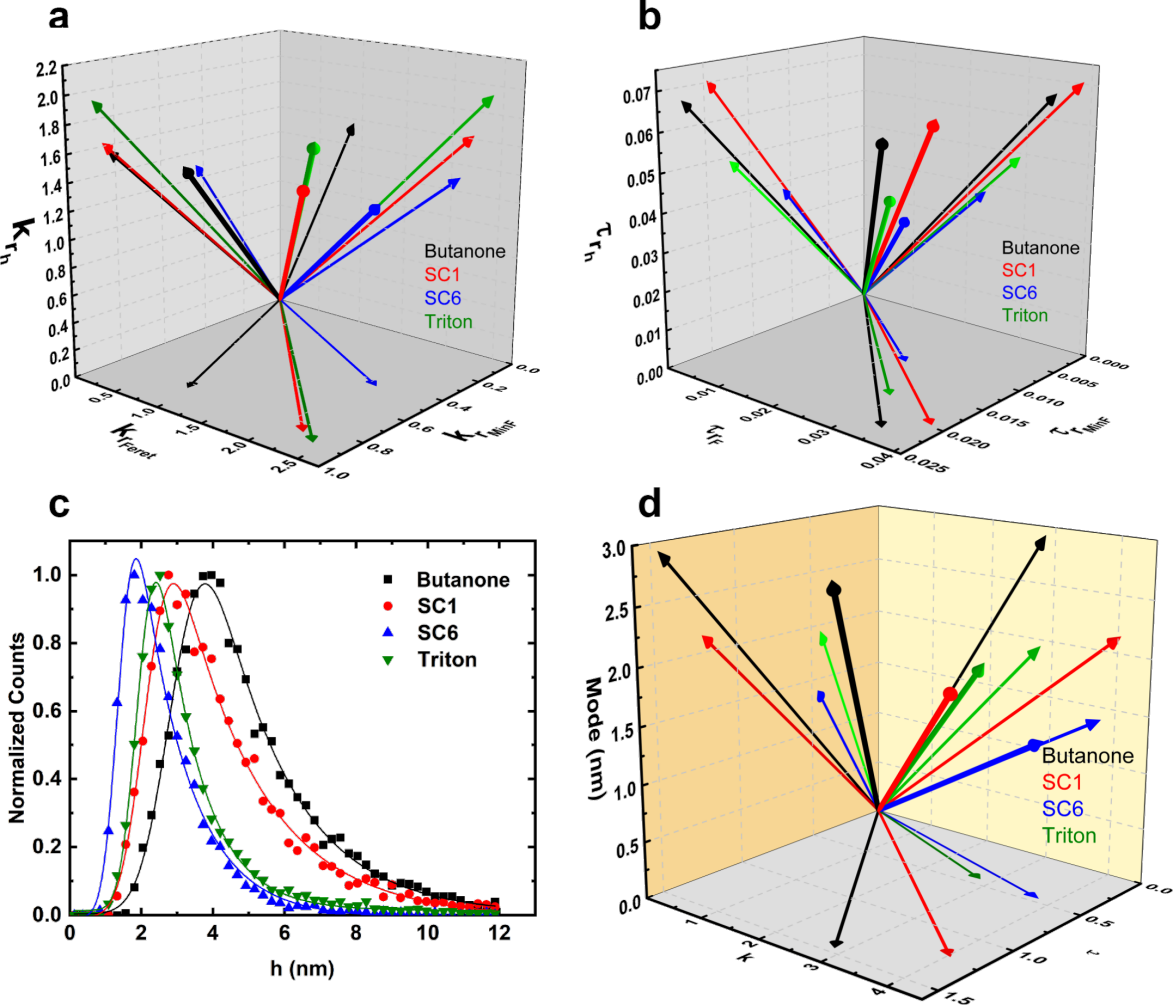
Shape vectors *k* and τ for each sample and normalized distribution of flake thickness. (a) Shape vectors *k* (each dimension is related to the asymmetry of the distribution of the natural logarithm of one of the three dimensionless shape parameters *r*_h_, *r*_Feret_, and *r*_MinF_). (b) Trimness shape vectors τ for all four samples. (c) Normalized histogram of the flake thickness *h* (black squares: butanone; red circles: sodium cholate at 1 mg/mL; blue upward triangles: sodium cholate at 6 mg/mL; green downward triangles: Triton-X100). (d) 3D vector plot of three parameters obtained from the EMG fit adjusted to the *h* data in (c): mode, *k*, and τ.

An in-depth discussion of these can be found elsewhere [25,29]. Figure 4a,b displays the topological vectors constructed for each sample for *k* and τ. Starting with the *k* shape vectors, the most symmetric distribution regarding *r*_h_ (thickness) was observed for the SC6 sample. Butanone and SC1 samples have a very similar asymmetry and Triton-X100 is the most asymmetrical of all four samples (thicker flakes cause the tail of the distribution to be more prominent). Exfoliation in butanone also results in a more symmetric distribution of the *r*_Feret_ parameter and again, SC1 and Triton-X100 samples are very similar, with SC6 being the most asymmetrical of all four samples. Regarding the distribution of *r*_MinF_, in contrast, the SC6 sample is the most symmetrical.

For the trimness parameter, τ (Figure 4b), a small value indicates a more symmetric and narrow distribution. The SC6 sample has the most trimmed distribution for all dimensionless shape parameters of all four samples. SC1 and butanone exhibit similar values (less trimmed) and Triton-X100 lies in between.

Finally, let us add to the initial flake size discussion by analyzing the flake thickness distribution instead of the dimensionless parameters. The normalized histograms of flake thickness for all four samples (Figure 3c) are well adjusted by the EMG probability density function (Equation 3). The thickness distribution is clearly asymmetric. Thicker flakes resulting in the tail of the curve have not been removed by the low-acceleration centrifugation. The mean flake thickness values have the same trend as the mean values displayed in Table 2: *h*_SC6_ < *h*_Triton-X100_ < *h*_SC1_ < *h*_butanone_. For the SC6 sample, the most common flake would be a bilayer talc flake.

For symmetry analysis, Figure 4d adds to what can be seen in Figure 4c with vectorial representation of mode, *k*, and τ for the *h* distribution of all four samples. Triton-X100 and SC6 have the most trimmed distributions while the most symmetrical are those of butanone and Triton X-100. All comparisons can be found in Table 3.

**Table 3:** Summary of the analysis for all four samples.

Parameter	Meaning	Order

mean flake thickness	lower values indicate thinner flakes	SC6 < Triton-X100 < SC1 < butanone
*M*/*m*	lower values indicate that fewer small flakes are needed to account for the mass of a large bulky flake	SC1 < butanone < Triton-X100 < SC6
*k* *_rh_*	lower values indicate more symmetrical distributions	SC6 < butanone < SC1 < Triton-X100
*k* *_r_*_Feret_	lower values indicate more symmetrical distributions	butanone < SC6 < SC1 < Triton-X100
*k* *_r_*_MinF_	lower values indicate more symmetrical distribution	SC6 < butanone < SC1 < Triton-X100
τ*_rh_*	lower values indicate more trimmed distribution	SC6 < Triton-X100 < butanone < SC1
τ*_r_*_Feret_	lower values indicate more trimmed distribution	SC6 < Triton-X100 < butanone < SC1
τ*_r_*_MinF_	lower values indicate more trimmed distribution	SC6 < Triton-X100 < SC1 < butanone
*k* *_h_*	lower values indicate more symmetrical distribution	Triton-X100 < butanone < SC6 < SC1
τ*_h_*	lower values indicate more symmetrical distribution	Triton-X100 < SC6 < SC1 < butanone

Finally, let us analyze advantages and drawbacks of each sample. An aqueous solution of 6 mg/mL sodium cholate is a widely recommended medium for liquid exfoliation [6,11]. For talc, it does yield the sample with the lowest average flake thickness (2.7 nm). However, it has the largest *M*/*m* ratio. The flakes are usually very small. Thus, many small flakes are required to compensate for the mass of rare but existing larger flakes. This means that while the number of thin flakes greatly exceeds the number of bulky ones, the mass of the latter is considerably large. Regarding the symmetry of the dimensionless shape parameters, the flakes of the SC6 sample are the most symmetrical, except for *r*_Feret_, for which they are the second most symmetric. The SC6 flakes are also the ones with the most prominent 2D shape, that is, the flakes are more plate-like than those of all other samples. All this makes this sample appropriate for applications in which monolayers and bilayers are required and flakes are all similar in a narrow range of 2D shapes.

The SC1 sample with more water added to the sample (sodium cholate and talc concentrations are reduced to 1 mg/mL) makes the average flake thickness increase to 4.5 nm. Also, it drastically brings down the *M*/*m* ratio (by more than seven times). It also makes the distribution of shape and size parameters less symmetrical. Nevertheless, this sample is well suited for applications that require thin flakes (mostly not a single layer) and the overall mass of talc to be mostly constituted of thin flakes. It should be stressed that SC is very difficult to remove from the exfoliated sample and applications using this solution must tolerate SC residues.

To avoid exfoliation medium residues, organic solvents of low boiling points could be used. Butanone was tested here and yields the largest mean flake thickness (5.2 nm). This is almost the double that of the SC6 sample, but it is still in the few-layer range. The *M*/*m* ratio is the second smallest, indicating that most of the mass of the sample is from few-layer flakes. The distribution of the shape parameters is fairly symmetric compared to the other samples but not very trimmed. The flakes are the most 3D-like ones, meaning they are more voluminous than other samples. Overall, if removability of the extraction medium is critical and few layers are required without the need for most flakes being monolayers, butanone is a good option.

Triton X-100 is also a widely employed surfactant for LPE. Since it is a nonionic surfactant, it is compatible with materials with surface charges. It is less expensive than SC and yields a sample with an average thickness of 3.4 nm, the second lowest one. Its *M*/*m* ratio is only smaller than that of the SC6 sample. However, it is about 2.4 times smaller, making it a good candidate for applications that require few-layer flakes predominating in number and in mass at the same time. It is the least symmetrical sample regarding the shape parameters, but it is fairly trimmed and the most symmetrical in flake size distribution.

## Conclusion

A thorough characterization of flake size and shape was performed for samples of liquid-phase exfoliated talc in four different media. LPE is a robust, scalable production route to obtain 2D nanomaterials from minerals. However, many parameters need to be adjusted to obtain a product suitable for a given application. Here, the choice of the medium was addressed while other parameters (mechanical energy source, exfoliation time, centrifugation acceleration and duration, and sample deposition) were kept constant. Four different media were employed to exfoliate talc. Aqueous solutions of sodium cholate at 1 and 6 mg/mL (with the talc powder concentration adapted to keep the cholate/talc ratio constant), an aqueous solution of Triton-X100, and pure butanone.

The exfoliation medium has an influence on flake size and shape and should be chosen according to the desired application. Implications go beyond the mean number of layers of the flakes (all four media yielded few-layer-rich solutions). Flake size (variance and asymmetry of distribution), few-layer-to-bulk mass ratio, and 1D/2D/3D shape characteristics also varied.

Table 3 gives a summary of the efficiency of each medium in producing flakes with the listed size and shape features. Our procedure puts to use previously published flake analysis methodology, highlighting the importance of obtaining information on thousands of flakes and using appropriate statistical descriptions to analyze the data.

## Experimental

**Materials.** Talc was obtained through a donation of a sample from Minas Gerais state, Brazil. X-ray diffraction (XRD) was performed to characterize the sample composition. The rock was manually milled to a fine powder. Sodium cholate and Triton-X100 were purchased from Sigma-Aldrich and used as received. All organic solvents were of analytical grade and used as received. Deionized water (resistivity 18.2 MΩ·cm) from a milliQ system was used for solution preparation. AFM measurements were performed on silicon substrates with an oxide layer, Si/SiO*_x_*. Substrates were functionalized with (3-aminopropyl)triethoxysilane (APTES) following the procedure reported by Fernandes and co-workers [24].

**X-ray diffraction.** XRD was performed in a Rigaku Geigerflex 2037 diffractometer with a graphite monochromator using Cu Kα radiation (1.54056 Å) in the Bragg–Brentano geometry (θ/2θ).

**Talc liquid-phase exfoliation.** Before submitting the material to the liquid exfoliation process, a purification step was performed to remove any contaminations [11]. Talc powder was sonicated for 1 h in chloroform and then the solution was left to decant. The supernatant was discarded, and the process was repeated with acetone and water. Finally, the powder was collected and dried for 12 h at 60 °C in an oven. The purified talcum powder was placed in an aqueous solution of the surfactant of choice (or pure butanone) (Table 1). For sodium cholate (SC), 6 mg/mL (concentrated) and 1 mg/mL (diluted) solutions in DI water were prepared. Talcum powder was added to the surfactant solutions at 1 mg talc to 1 mL of diluted SC solution and 6 mg talc to 1 mL of concentrated SC solution. Triton-X100 solutions were 1 mg/mL. Butanone was used pure as received. For Triton-X100 and butanone, talc was added at 6 mg/mL. Glass vials containing the solutions were placed in an ultrasonic bath (Elma, S10H) for 15 h. The water bath temperature was monitored and controlled by adding ice to keep it below 40 °C if required. The resulting solutions were centrifuged at 1000*g* for 1 h (Multifuge X3R Thermo Scientific) to remove non-exfoliated material [26]. All analyses were performed with the collected supernatant. Purified talc powder of the same batch was used to prepare different medium samples to ensure the starting material was the same. All exfoliation parameters and material were kept as equal as possible to ensure that the differences of the flakes were associated to the medium influence and not to any other parameter.

**AFM measurements.** Sample preparation for AFM measurements followed the procedure designed by Fernandes et al. [24] and Santos and co-workers [25]. In short, a solution (1:40) of APTES in DI water was prepared. Si/SiO*_x_* substrates were immersed in the solution for 15 min. Subsequently, each substrate was rinsed with DI water and blown dry with pure N_2_ five times to ensure the removal of any residual APTES molecules. This step is crucial to ensure that talc flakes of all sizes adhere to the substrate and do not stack. Talc deposition is achieved employing spread coating of the solution onto the functionalized substrate. A drop that covers all the substrate is deposited on the surface and allowed to be in contact with it for 30 to 60 s to ensure optimal coverage. Then the sample is rinsed again in DI water to remove loose flakes and residual surfactant. An in-depth discussion of this procedure can be found in [24].

AFM measurements were performed on a Park XE-70 microscope, in intermittent contact mode using commercial silicon probes (MikroMasch, HQ:NSC35/AlBs or HQ:NSC36/AlBs). For each sample, nine different 5 μm × 5 μm fields were chosen at random and scanned at 0.5 Hz with 500 pixels/line (lateral resolution of 10 nm/pixel). Image processing (line and plane corrections) and flake counting [24] was performed using Gwyddion [30] and ImageJ software, respectively. The standard particle analysis toolbox available in ImageJ was employed to obtain the flake dimensions.
